# Stability Analysis of a Model for Foreign Body Fibrotic Reactions

**DOI:** 10.1155/2012/809864

**Published:** 2012-09-13

**Authors:** A. Ibraguimov, L. Owens, J. Su, L. Tang

**Affiliations:** ^1^Department of Mathematics and Statistics, Texas Tech University, P.O. Box 41042, Lubbock, TX 79409-1042, USA; ^2^Department of Mathematics, The University of Texas at Arlington, Arlington, TX 76019, USA; ^3^Department of Bioengineering, The University of Texas at Arlington, Arlington, TX 76019, USA

## Abstract

Implanted medical devices often trigger immunological and inflammatory reactions from surrounding tissues. The foreign body-mediated tissue responses may result in varying degrees of fibrotic tissue formation. There is an intensive research interest in the area of wound healing modeling, and quantitative methods are proposed to systematically study the behavior of this complex system of multiple cells, proteins, and enzymes. This paper introduces a kinetics-based model for analyzing reactions of various cells/proteins and biochemical processes as well as their transient behavior during the implant healing in 2-dimensional space. In particular, we provide a detailed modeling study of different roles of macrophages (MΦ) and their effects on fibrotic reactions. The main mathematical result indicates that the stability of the inflamed steady state depends primarily on the reaction dynamics of the system. However, if the said equilibrium is unstable by its reaction-only system, the spatial diffusion and chemotactic effects can help to stabilize when the model is dominated by classical and regulatory macrophages over the inflammatory macrophages. The mathematical proof and counter examples are given for these conclusions.

## 1. Introduction

Recently, intensive research efforts have been focusing on developing mechanistic computational models for wound healing related processes. Wound healing is a very complicated biochemical and biophysical phenomenon, with many facets and subprocesses, including the inflammatory response process, angiogenesis as well associated fibrotic reactions. Many cells, enzyme, growth factors, and proteins participate at different stages of the wound healing reactions, and they form a network of signaling pathways that in turn leads to inflammatory, angiogenesis, and fibrotic reactions. We refer to the review by Diegelmann and Diegelmann and Evans 2004 [1] for a brief review of the recent scientific work.

As a subarea of general wound healing research, healing processes involved in medical implantations are of significant application for modern medicine [2–4]. It is commonly accepted that implants may cause foreign body reactions that are initiated with implant-mediated fibrin clot formation, followed by acute inflammatory responses [4, 5]. The inflammatory chemokines released by adherent immune cells serve as strong signals for triggering the migration of macrophages and fibroblasts from the surrounding tissues and circulation toward the implant surface [5]. The implant-recruited fibroblasts consequently synthesize chains of amino acids called procollagen, a process that is activated by growth factors, including in particular type-*β* transforming growth factor (TGF-*β*) [6, 7] to become collagen, the dominant ingredient of the extracellular matrix (ECM) [8]. These processes may, however, differ slightly between dermal wound healing and implantation when it comes to specific activation and inhibition loops of reactions.

Among inflammatory cells, macrophages (*M*Φ) are found to reside in the wound [9]. The roles of macrophages are multiple and stand prominent in the activations and inflammations during implantation. *M*Φ are known to remove damaged tissue and foreign debris via phagocytosis. In addition, *M*Φ often release a variety of chemokines to recruit other cell types, such as fibroblasts, which participate in the remodeling of ECM. The specific roles of *M*Φ vary significantly at different stages of healing process. Work by Mosser and Edwards 2008 [10] has shown there to be at least 3 phenotypes of *M*Φ, each of which displays a different functionality. Classically activated *M*Φ represent the effector *M*Φ that are produced during cell-mediated immune responses. Two signals, interferon-*γ* and tumor-necrosis factor-*α*, give rise to these effector *M*Φ which have enhanced microbicidal or tumoricidal capacity and secrete high levels of proinflammatory cytokines and mediators. Assisted in part by the production of transforming growth factor type *β* (TGF-*β*), the clearance of apoptotic inflammatory, as well as noninflammatory cells by classical *M*Φ, can lead to an inhibition of inflammation [11, 12]. Wound healing *M*Φ (or inflammatory *M*Φ) can develop in response to innate or adaptive signals through interleukin-4. In turn, interleukin-4 stimulates arginase activity in *M*Φ, allowing them to convert arginine to ornithine, a precursor of polyamine and collagen, thereby contributing to the production of extracellular matrix (ECM) [13]. Regulatory *M*Φ can also arise during the later stages of adaptive immune responses, the primary role of which dampen, the immune response and limits inflammation through production of interleukin-10 [14]. Although all three phenotypes were observed experimentally within the dermal wound healing context, the phagocyte biomaterial interactions are known to be similar here for foreign body reactions.

While experiments are still the main stay in the studying of wound healing related process, significant progress has also been made in detail predictive modeling based on biochemical and biophysics principles. For dermal wound healing, basic reactions were first considered in studies by Dale et al. 1996 [15], 1997 [16]; Dallon et al. 2001 [17] and many others. Their models incorporated the key features of kinetics which are essential to dermal wound healing. Their results have successfully described the dynamics and compare favorably with experiments, in terms of healed ECM fiber ratio, spatial orientation, and other features. Recently, the work of Schugart et al. 2008 [18] and Xue et al. 2009 [19] further included angiogenesis equations to the healing process and examined the positive effects of increased oxygen level in accelerating the healing and closure of open wound, suggesting new insights for the healing. Furthermore, a wound healing model based more on cell migration was considered in Arciero et al. 2011 [20].

Inflammatory reactions are important to wound healing as they activate many key agents for the healing process, however, prolonged inflammation may cause excessive scars and chronic wounds. Through interactions between immune mediators, phagocytes in the blood and tissue, the acute inflammatory response was modeled and analyzed by reduced compartmental models in Reynolds et al. 2006 [21] and Day et al. 2006 [22]. Closely related to dermal wound healing and implantation, atherogenesis in blood vessels was modeled by continuum equations in Ibragimov et al. [23]. The concept of debris and phagocytosis in [21, 22, 24] is analogous to our current model, which assumes that the digestion of dead cells (or tissues) initiates the entire healing process. Further, addition of stem cells can create a new dimension to the healing and implantation process; we mention Lemon et al. 2009 [24] for their new mathematical tool in providing quantitative analysis for this growing field.

Our primary goal in this paper is to use computational modeling to study the fibrotic reaction process following implantation with specific attention given to the effects caused by varying the mix of different phenotypes of *M*Φ. Our modeling results indicate trends for these variations, serving as a plausible clue for developing new experiments.

The main mathematical contribution of this paper is as follows. The nonzero equilibrium of our model represents an inflamed state. If it is linearly stable in terms of the corresponding ODE system (the reactions network of the model), then it is also stable for the full system (which includes spatial diffusion and chemotaxis). In other words, spatial effects cannot destabilize the equilibrium if it is stable in its pure reactions. However, even if the equilibrium is unstable by its reaction system, the spatial diffusion and chemotactic effects can help to stabilize the equilibrium under several conditions. These conditions suggest the need for the model to be dominated by classical and regulatory macrophages over the inflammatory macrophages. The mathematical proof and counter examples are given for these results.

We organize the paper as follows. In Section 2, we introduce the model and the modeling considerations. In Section 3, we discuss the spatially uniform equilibria and their stability in relation to the ODE system representing the reaction system without spatial variations. In Section 4, we prove that if the equilibrium is stable in ODE sense, then it is stable for the full system with respect to any small spatial perturbation measured in *L* ^2^. In Section 5, we provide a set of sufficient conditions for the equilibrium to be stable for the full system, which allows us to explicitly give a counter example where PDE solutions can be conditionally stable without requiring stability in the ODE system. A brief summary and discussion are presented in Section 6.

## 2. Modeling Based on Chemical Kinetics Equations

Our foreign body reaction model is partially from the mass-action kinetics framework developed by Schugart et al. 2008 [18], which modeled wound healing under oxygen pressure. In medical implantation processes, new kinetics of *M*Φ reactions were added in the framework. The main biological question that we hope to address is the variance between tissue responses at different percentages of classical, inflammatory, and regulatory *M*Φ cells during foreign body fibrotic reaction processes. We model the following:

(1) $$\begin{array}{c} \frac{\partial D}{\partial t}=D_{d}\nabla^{2}D-f_{0}\lambda_{1}MD+{\tilde{f}}_{0}\lambda_{3}M, \end{array}$$

(2) $$\begin{array}{c} \frac{\partial C}{\partial t}=D_{c}\nabla^{2}C+f_{1}D+f_{2}\lambda_{3}M-f_{3}\lambda_{2}MC-f_{4}C, \end{array}$$

(3) ∂F∂t=Df∇2F−χ0∇·(F∇C)+a1λ1M +a2F(a2−a3a2−FF0)+a12CFH(F0−F),

(4) ∂M∂t=Dm∇2M−χ1∇·(MH(M0−M)∇C) −a0M+a11CMH(M0−M),

(5) $$\begin{array}{c} \frac{\partial E}{\partial t}=D_{e}\nabla^{2}E-\nabla \cdot \Phi +a_{16}F\left(1-\frac{E}{E_{0}}\right), \end{array}$$

where ∇^2^ = ∇·∇, and the vector field

(6) $$\begin{array}{c} \Phi =\frac{BD_{f}}{F_{0}}E\nabla F+\frac{B\chi_{j}}{F_{0}}\left(EFH\left(F_{0}-F\right)\nabla C\right), \end{array}$$

and all coefficients are positive. The form of the logistic terms in (3) is for representing biological meanings of the coefficients.

In the system (1)–(5), the debris cell population *D* represents dead tissue cells following implantation. Abnormal white blood cells and molecules caused by the surgery are also included in this debris term, which is assumed to be the initiation point of reactions. We assume that they are digested by *M*_1_-classical *M*Φ and that *M*_3_-inflammatory *M*Φ contribute to the accumulation of debris during the healing process (as modeled in (1)).

The chemoattractant consists mainly of various forms of growth factors including tissue growth factors type *β* (TGF*β*) released during the tissue injury. The chemoattractant field *C* is enhanced by the presence of debris and *M*_3_-inflammatory *M*Φ cell, but is inhibited by *M*_2_-regulatory *M*Φ cells. In (2), we assume that cell spatial migration occurs through diffusion and chemotactic migration based on the gradient field of *C*.

Fibroblast density *F* represents a main cell type in secreting collagen (a major component of ECM). Fibroblast proliferation and collagen synthesis are upregulated by the chemoattractant gradient field *C*. Thus fibroblast population *F* (shown in (3)) can be approximated by a chemically enhanced logistic growth *F*(1 − (*F*/*F*_0_)) with a threshold *F*_0_, along with its diffusion in space modeled by *D*_*f*_∇^2^ *F*, chemotactic migration by −*χ*_0_∇·(*F*∇*C*) and its natural decay according to time as shown in (3). New experimental data also shows autocrine upregulation of fibroblast by TGF*β* without chemotaxis [25]; this effect is also included in the modeling. The term *a*_3_ *F* is the decaying factor.

Macrophage density, *M*, is the summation of *M*_1_-classical *M*Φ, *M*_2_-regulatory *M*Φ, and *M*_3_-inflammatory *M*Φ. We assume that they each take on a proportion *λ*_1_, *λ*_2_, and *λ*_3_ of *M*Φ, respectively. Each phenotype *M*_*j*_, *j* = 1,2, 3, may take a different share of *M*Φ at different stages of foreign body fibrotic reactions. However, our model simplifies the situation in that (a) the proportions *λ*_1_, *λ*_2_, and *λ*_3_ for different phenotypes of *M*Φ are fixed, and (b) the total *M*Φ population is set to share one common biochemical reaction equation (4), since its basic biochemical properties are similar. The proliferation of *M*Φ at the field is through diffusion and migration upregulated by the chemotactic gradient field *C*, but the production does reach a limiting value once the *M*Φ population reaches its saturation of *M*_0_. *M*Φ cell apoptosis and proliferation caused by the direct interaction with chemoattractants are also assumed.

Finally in (5), fibroblasts secrete procollagen which is then activated by the chemoattractant TGF*β*s into collagen (or ECM) represented by the quantity *E*. We also incorporate the effects of ECM diffusion, fibroblast movement, chemotactic migration, and ECM saturation in mass-action law. In all discussions, *H* is the Heaviside function, and *M*_0_ is the *M*Φ saturation level.

We assume in our implant model that the computational domain is large enough and also the cell changes are slow enough (measured in days) that there is no significant boundary flux, allowing us to take homogeneous Neumann boundary conditions as a reasonable approximation.

**Definition 1**

Let us define inflammatory equilibrium as a strictly nonzero constant vector *U*_*e*_ in 5-dimensional space *U*_*e*_ = (*d*_*e*_, *c*_*e*_, *f*_*e*_, *m*_*e*_, *e*_*e*_) with *d*_*e*_ > 0, *c*_*e*_ > 0, *F*_*o*_ ≥ *f*_*e*_ > 0, *M*_*o*_ ≥ *m*_*e*_ > 0, *e*_*e*_ = *E*_0_ > 0, which solves system of the equations (1)–(5).

**Remark 2**

In the case of a no-flux boundary condition, the spatially uniform steady state is often used when modeling inflammatory response in tissue (see e.g., [23, 26] and reference therein). A physically realistic, nonnegative set of equilibriums can easily be obtained by letting the RHS of the original system (1)–(5) equal to zero. It is natural to define the trivial (zero) equilibrium as ground or healthy state and study its stability. Instability of the ground state is usually interpreted as unfavorable development of the disease. In this paper we take a different approach and are interested in analyzing the stability of the *abnormal/inflammatory* equilibrium which is nonzero for all five components of the unknown. This equilibrium can be stable or unstable depending on the parameters of the model. In this case, instability of the equilibrium does not necessarily mean an unhealthy response of the immune system. An instability of a nonzero equilibrium can lead to a ground healthy state (best case scenario), to another steady state (uncertain developments), or to infinity (acute development). If in contrary, the perturbation of *U*_*e*_ is linearly stable and vanishes at time infinity, then *U*_*e*_ can be interpreted as sustainable. All these make linear stability analysis very appealing from both a theoretical and applied point of view. It is worth mentioning that from a biological point of view, a strictly positive steady state *U*_*e*_ can be transitioned from some other nonstrictly positive state. We believe that this type of interpretation of the inflammatory equilibrium stability conditions is logical and presents an example of a sustainable wound which does not heal over the course of a long time period (see [19–21]). An indirect analogy of such an inflammatory (chronically) stable equilibrium has been introduced and applied for studying biological dynamic system in virology for some years (see e.g., [27]). At this stage of the research, we are studying stability of the strictly positive state *U*_*e*_ mostly as a model of inflammatory equilibrium, without analysis of its genesis. As commonly occurs in biomedical research, the mathematical model can often provide nonintuitive insights into dynamics of inflammatory responses in the wound healing processes and can suggest new avenues for experimentation. In the forthcoming sections, sufficient conditions on the parameters of the system of the equation guarantee stability of nonzero equilibrium.

### 2.1. Linearized System

Let perturbation near this equilibrium be as following:

(7) $$\begin{array}{c} d=D-d_{e},\;c=C-c_{e},\;f=F-f_{e},\\ m=M-m_{e},\;e=E-e_{e}. \end{array}$$

Denote vector field of the perturbation by $\overset{-}{v}\left(x,t\right)=\left(d,c,f,m,e\right)$. Then the linearized system for $\overset{-}{v}\left(x,t\right)$ will take the following form:

(8) $$\begin{array}{c} \frac{\partial d}{\partial t}=D_{d}\nabla^{2}d-b_{11}d-b_{14}m\\ \frac{\partial c}{\partial t}=D_{c}\nabla^{2}c-b_{21}d-b_{22}c-b_{24}m,\\ \frac{\partial f}{\partial t}=D_{f}\nabla^{2}f-\chi_{f}\nabla^{2}c-b_{32}c-b_{33}f-b_{34}m,\\ \frac{\partial m}{\partial t}=D_{m}\nabla^{2}m-\chi_{m}\nabla^{2}c-b_{42}c-b_{44}m,\\ \frac{\partial e}{\partial t}=D_{e}\nabla^{2}e-\chi_{e1}\nabla^{2}f-\chi_{e2}\nabla^{2}c-b_{53}f-b_{55}e. \end{array}$$

Here,

(9) χf=feχ0, χm=χ1me, χe1=BDfe0F0, χe2=Bχje0feF0,b11=f0λ1me, b12=0, b13=0,b14=−(f~0λ3−f0λ1de), b15=0,b21=−f1, b22=f3λ2me+f4,b24=−(f2λ3−f3λ2ce), b23=b25=0,b31=0, b32=−a12fe,b33=−[a2(1−2feF0)+a12ce−a3],b34=−a1λ1, b35=0,b41=0, b42=−a11me, b43=0,b44=a0−a11me, b45=0,b51=0, b52=0, b53=−a16(1−eeE0),b54=0, b55=a16feE0.

## 3. Spatially Uniform Equilibrium States and Linear Stability in ODE System

We now focus on equilibrium states that are uniform in space for this Neumann problem. By removing the spatial variations, (1)–(5) reduce to the following ODE system:

(10) $$\begin{array}{c} \frac{dD}{dt}=-f_{0}\lambda_{1}MD+{\tilde{f}}_{0}\lambda_{3}M,\\ \frac{dC}{dt}=f_{1}D+f_{2}\lambda_{3}M-f_{3}\lambda_{2}MC-f_{4}C,\\ \frac{dF}{dt}=a_{1}\lambda_{1}M+a_{2}F\left(1-\frac{F}{F_{0}}\right)-a_{3}F+a_{12}CFH\left(F_{0}-F\right),\\ \frac{dM}{dt}=-a_{0}M+a_{11}CMH\left(M_{0}-M\right),\\ \frac{dE}{dt}=a_{16}F\left(1-\frac{E}{E_{0}}\right). \end{array}$$

In looking for the equilibrium of the simplified system, (10), we assume that our values are taken to be below threshold and therefore we ignore the Heaviside functions. There are several possible equilibrium states, but as it was pointed out earlier, we focus on what one can call the interior equilibrium, one in which none of the components of the equilibrium are zero. We let the right-hand side of (10) to be zero. After some algebraic work, one can obtain the following explicit formula for a unique, nonzero solution *U*_*e*_ = (*d*_*e*_, *c*_*e*_, *e*_*e*_, *m*_*e*_, *f*_*e*_):

(11) $$\begin{array}{c} d_{e}=\frac{{\tilde{f}}_{0}\lambda_{3}}{f_{0}\lambda_{1}},\\ c_{e}=\frac{a_{0}}{a_{11}},\\ e_{e}=E_{0},\\ m_{e}=\frac{f_{4}f_{0}\lambda_{1}a_{0}-a_{11}f_{1}{\tilde{f}}_{0}\lambda_{3}}{f_{0}\lambda_{1}\left(f_{2}\lambda_{3}a_{11}-f_{3}a_{0}\lambda_{2}\right)},\\ f_{e}=\left[\frac{F_{0}}{2a_{2}}\right]\left[\left(a_{2}-a_{3}+a_{12}\left(\frac{a_{0}}{a_{11}}\right)\right)+L_{1}\right]. \end{array}$$

Here, $L_{1}=\sqrt{{\left(a_{2}-a_{3}+a_{12}\left(a_{0}/a_{11}\right)\right)}^{2}+4\left(a_{2}/F_{0}\right)a_{1}\lambda_{1}m_{e}}$.

**Remark 3**

In order for the inflammatory equilibrium to exist, it is necessary and sufficient that macrophage percentages satisfy the following:

(12) $$\begin{array}{c} \frac{f_{4}f_{0}\lambda_{1}a_{0}-a_{11}f_{1}{\tilde{f}}_{0}\lambda_{3}}{\left(f_{2}\lambda_{3}a_{11}-f_{3}a_{0}\lambda_{2}\right)}>0, \end{array}$$

requiring either

(13) $$\begin{array}{c} f_{2}\lambda_{3}a_{11}>f_{3}a_{0}\lambda_{2},\;\;f_{4}f_{0}\lambda_{1}a_{0}>a_{11}f_{1}{\tilde{f}}_{0}\lambda_{3}, \end{array}$$

or

(14) $$\begin{array}{c} f_{2}\lambda_{3}a_{11}<f_{3}a_{0}\lambda_{2},\;\;f_{4}f_{0}\lambda_{1}a_{0}<a_{11}f_{1}{\tilde{f}}_{0}\lambda_{3}.\;\; \end{array}$$

**Figure 1:**
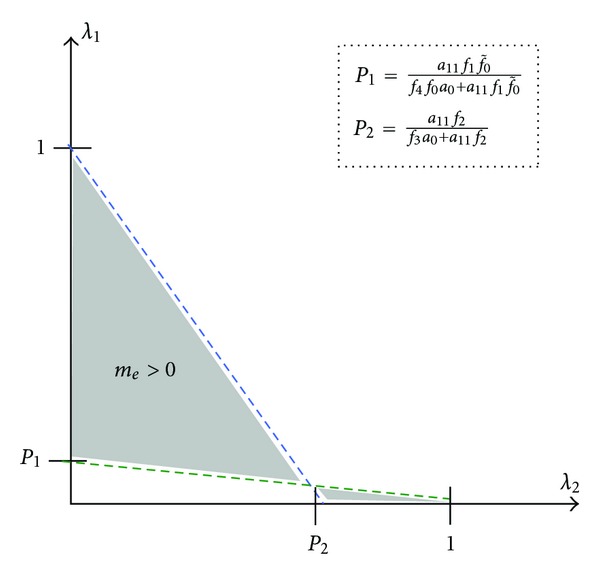
Illustration of the parameter range to ensure that *m*_*e*_ > 0.

Condition on the parameters in (12) says that inflammatory macrophages dominate over either regulatory or classical macrophages and are guaranteeing existence of the inflamed steady state. This point will be expounded on further in the analysis of the conditions for stability of the nonzero equilibrium state. The illustration (Figure 1) provides a visualization of the necessary macrophage phenotype parameter ranges. “Hereafter we assume that the parameters of the original model satisfy condition (12).”

Turning now to satisfy the stability of the system at the equilibrium, we find the linearized system to be as follows:

(15) dddt=−b11d−b14m,dcdt=−b21d−b22c−b24m,dfdt=−b32c−b33f−b34m,dmdt=−b42c−b44m,dedt=−b53f−b55e,

where

(16)     b11=f0λ1me,  b14=−(f~0λ3−f0λ1de),b21=−f1,  b22=f3λ2me+f4,b24=−(f2λ3−f3λ2ce),    b32=−a12fe,  b33=−[a2(1−2feF0)+a12ce−a3],    b34=−a1λ1,    b42=−a11me,  b44=a0−a11ce,    b53=−a16(1−eeE0),  b55=a16f0E0.

Equations (32)–(39) in matrix form yields as follows:

(17) $$\begin{array}{c} {\left[\begin{array}{c} d\\ c\\ f\\ m\\ e \end{array}\right]}^{\prime }=-B\left[\begin{array}{c} d\\ c\\ f\\ m\\ e \end{array}\right], \end{array}$$

where **B** is:

(18) $$\begin{array}{c} B=\left[\begin{array}{ccccc} b_{11} & 0 & 0 & b_{14} & 0\\ b_{21} & b_{22} & 0 & b_{24} & 0\\ 0 & b_{32} & b_{33} & b_{34} & 0\\ 0 & b_{42} & 0 & b_{44} & 0\\ 0 & 0 & b_{53} & 0 & b_{55} \end{array}\right]. \end{array}$$

For stability analysis, we look at the eigenvalues of matrix −**B**; for convenience, we rearrange our equations in the following form:

(19) $$\begin{array}{c} {\left[\begin{array}{c} m\\ d\\ c\\ f\\ e \end{array}\right]}^{\prime }=\left[\begin{array}{ccccc} -b_{44} & 0 & -b_{42} & 0 & 0\\ -b_{14} & -b_{11} & 0 & 0 & 0\\ -b_{24} & -b_{21} & -b_{22} & 0 & 0\\ -b_{34} & 0 & -b_{32} & -b_{33} & 0\\ 0 & 0 & 0 & -b_{53} & -b_{55} \end{array}\right]\left[\begin{array}{c} m\\ d\\ c\\ f\\ e \end{array}\right]. \end{array}$$

We break −**B** into a 3-block and a 2-block as follows:

(20) $$\begin{array}{c} -B_{1}=\left[\begin{array}{ccc} -b_{44} & 0 & -b_{42}\\ -b_{14} & -b_{11} & 0\\ -b_{24} & -b_{21} & -b_{22} \end{array}\right],\;\;-B_{2}=\left[\begin{array}{cc} -b_{33} & 0\\ -b_{53} & -b_{55} \end{array}\right]. \end{array}$$

Since det(−**B** − *σ* **I**) = det(−**B**_1_ − *σ* **I**) det(−**B**_2_ − *σ* **I**), we find the eigenvalues by looking at the eigenvalues of the 3-block, −**B**_1_, and the two block, −**B**_2_, separately. We also simplify by noting that with the equilibrium values found above, *b*_44_ = 0 and *b*_14_ = 0 s.t.

(21) det⁡[−B1−σI]=[−σ0−b420−b11−σ0−b24−b21−b22−σ]=−σ(b11+σ)(b22+σ)+b24(b42(b11+σ))=−(b11+σ)(σ2+b22σ−b24b42),

solving for the roots we get the following eigenvalues:

(22) $$\begin{array}{c} \sigma_{1}=-b_{11}, \end{array}$$

(23) $$\begin{array}{c} \sigma_{2}=\frac{-b_{22}-\sqrt{{\left(b_{22}\right)}^{2}+4b_{42}b_{24}}}{2}, \end{array}$$

(24) $$\begin{array}{c} \sigma_{3}=\frac{-b_{22}+\sqrt{{\left(b_{22}\right)}^{2}+4b_{42}b_{24}}}{2}. \end{array}$$

The lower triangular −**B**_2_ gives us our final two eigenvalues:

(25) $$\begin{array}{c} \sigma_{4}=-b_{33}, \end{array}$$

(26) $$\begin{array}{c} \sigma_{5}=-b_{55}. \end{array}$$

ODE stability requires real parts of the *σ*_1_,…, *σ*_5_ to be negative. In the next remark, stability criteria are formulated in terms of the parameters of the model.

**Remark 4**

Under the model assumptions we have

(27) $$\begin{array}{c} -b_{11}<0\;\;\;-b_{22}<0,\;b_{42}<0,\;-b_{55}<0. \end{array}$$

Therefore, *σ*_1_ < 0, *σ*_2_ < 0, and *σ*_5_ < 0. Next, if

(28) $$\begin{array}{c} b_{33}=a_{2}\;\left(1-2\frac{f_{e}}{F_{0}}\right)+a_{12}c_{e}-a_{3}>0, \end{array}$$

then *σ*_4_ < 0. Finally, because *b*_42_ < 0, real part of *σ*_3_ is negative if and only if

(29) $$\begin{array}{c} b_{24}=-\left(f_{2}\lambda_{3}-f_{3}\lambda_{2}c_{e}\right)>0. \end{array}$$

Assumptions in (28) and (29) have clear biological interpretation.

Condition *b*_33_ > 0 requires

(30) $$\begin{array}{c} \left[a_{2}\left(1-2\frac{f_{e}}{F_{0}}\right)+a_{12}c_{e}<a_{3}\right], \end{array}$$

suggesting the need for the logistic growth of fibroblasts combined with the direct proliferation resulting from the presence of chemoattractants to be overcome by the death rate of fibroblasts.

Condition *b*_24_ > 0 requires

(31) $$\begin{array}{c} f_{3}\lambda_{2}c_{e}>f_{2}\lambda_{3}, \end{array}$$

suggesting that stability is aided when the percentage of regulatory macrophages out-weighs the percentage of inflammatory macrophages.

Note that from a mathematical point of view, conditions in the form of a strict inequalities imply a stronger property of the solution, namely asymptotic stability of the equilibrium. Lyapunov stability follows from the less restrictive condition with nonstrict inequalities.

## 4. ODE Linear Stability Implies PDE Linear Stability

Since the interior equilibrium solution represents the inflammatory state, one of the more biologically relevant questions is whether some modifications of conditions can cause the reactions to be away from the ill state and return to healthy state. Typically, the competition between diffusion and chemotaxis can aid the instability by creating spatial disturbance. One of the surprising findings for this system, however, is that if the equilibrium is stable by pure reactions, then it is stable for the whole reaction-diffusion-chemotactic system.

To start, we let

(32) $$\begin{array}{c} \overset{-}{v}\left(x,t\right)=e^{\sigma t}{\phi_{\mu }}_{n}\left(x\right)\left(u_{1},\ldots ,u_{5}\right) \end{array}$$

to be a vector with unknown five components and function *ϕ*_*n*_(*x*) to be the *n*th eigenfunction for Laplace equation with respect to Neumann boundary conditions:

(33) $$\begin{array}{c} \Delta \phi_{n}\left(x\right)=-\mu_{n}\phi_{\mu_{n}}\;\text{inside}\;\text{domain}, \end{array}$$

(34) $$\begin{array}{c} \frac{\partial \phi_{\mu_{n}}}{\partial n}=0\;\mathrm{on}\;\text{the}\;\text{boundary}\;\text{of}\;\text{the}\;\text{domain}. \end{array}$$

Let us for simplicity assume that the domain is convex such that *μ*_*n*_ ≥ 0 for any *n* ∈ *ℕ* is an eigenvalue for the eigenvalue problem, and *ϕ*_*μ*_*n*__ is its corresponding eigenfunction. We will drop the subscripts *n* in the text below. Substituting the function $\overset{-}{v}\left(x,t\right)$ into equation one can get

(35) σu1=−Ddμu1−b11u1−b14u4,σu2=−Dcμu2−b21u1−b22u2−b24u4,σu3=−Dfμu3+χfμu2−b32u2−b33u3−b34u4,σu4=−Dmμu4+χmμu2−b42u2−b44u4,σu5=−Deμu5+χe1μu3+χe2μu2−b53u3−b55u5,

or

(36) $$\begin{array}{c} \left(\sigma +D_{d}\mu +b_{11}\right)u_{1}+b_{14}u_{4}=0,\\ b_{21}u_{1}+\left(\sigma +D_{c}\mu +b_{22}\right)u_{2}+b_{24}u_{4}=0,\\ \left(\sigma +D_{f}\mu +b_{33}\right)u_{3}-\left(\chi_{f}\mu -b_{32}\right)u_{2}+b_{34}u_{4}=0,\\ -\left(\chi_{m}\mu -b_{42}\right)u_{2}+\left(\sigma +D_{m}\mu +b_{44}\right)u_{4}=0,\\ -\chi_{e2}\mu u_{2}-\left(\chi_{e1}\mu -b_{53}\right)u_{3}+\left(\sigma +D_{e}\mu +b_{55}\right)u_{5}=0. \end{array}$$

Then in matrix form it takes a form

(37) $$\begin{array}{c} A\left(\sigma \right)\overset{-}{u}=0, \end{array}$$

with matrix *A* defined as follows:

(38) $$\begin{array}{c} \left(\begin{array}{ccccc} \left(\sigma +D_{d}\mu +b_{11}\right) & 0 & 0 & b_{14} & 0\\ b_{21} & \left(\sigma +D_{c}\mu +b_{22}\right) & 0 & b_{24} & 0\\ 0 & -\left(\chi_{f}\mu -b_{32}\right) & \left(\sigma +D_{f}\mu +b_{33}\right) & b_{34} & 0\\ 0 & -\left(\chi_{m}\mu -b_{42}\right) & 0 & \left(\sigma +D_{m}\mu +b_{44}\right) & 0\\ 0 & -\chi_{e2}\mu & -\left(\chi_{e1}\mu -b_{53}\right) & 0 & \left(\sigma +D_{e}\mu +b_{55}\right) \end{array}\right). \end{array}$$

Below, we will show that if the real part of all eigenvalues of matrix **B** is negative (corresponding ODE system is stable), then nontrivial solutions of (37) with parameter *σ* having negative real part exist. It is not difficult to see that the determinant of the matrix *A* has aform as follows:

(39) $$\begin{array}{c} P\left(\sigma \right)=\left(\;\sigma +D_{e}\mu +b_{55}\right)\left(\sigma \;+D_{f}\mu +b_{33}\right)\det\left(B_{1}\right). \end{array}$$

Here, *B*_1_ is a matrix associated to debris *u*_1_, chemotaxis *u*_2_, and macrophages *u*_4_ parameters only:

(40) $$\begin{array}{c} \left(\begin{array}{ccc} \left(\sigma +D_{d}\mu +b_{11}\right) & 0 & b_{14}\\ b_{21} & \left(\sigma +D_{c}\mu +b_{22}\right) & b_{24}\\ 0 & -\left(\chi_{m}\mu -b_{42}\right) & \left(\sigma +D_{m}\mu +b_{44}\right) \end{array}\right). \end{array}$$

Under the assumptions that the ODE part without diffusion is asymptotically stable, coefficients *b*_55_ and *b*_33_ should satisfy inequalities *b*_44_ = *b*_14_ = 0, *b*_55_ > 0 and *b*_33_ < 0.

We rearrange the matrix into a *u*_4_, *u*_1_, *u*_2_ order so that it is similar to the one addressed previously in the ODE stability analysis. Now,

(41) det⁡⁡[B1+σI]=[σ+Dmμ0b42−χmμ0b11+Ddμ+σ0b24b21b22+Dcμ+σ]=(σ+Dmμ)(b11+Ddμ+σ)(b22+Dcμ+σ) −b24(b42−χmμ)(b11+Ddμ+σ)=(b11+Ddμ+σ)(σ2+(b22+Dcμ+Dmμ)σ +Dmμ(b22+Dcμ)−b24(b42−χmμ)),

solving for the roots we get the following eigenvalues:

(42) $$\begin{array}{c} \sigma_{1}=-b_{11}-D_{d}\mu , \end{array}$$

(43) $$\begin{array}{c} \sigma_{3}=\frac{-\left(b_{22}+D_{c\mu }+D_{m\mu }\right)+\sqrt{{\left(b_{22}D_{c}\mu +D_{m}\mu \right)}^{2}+4\epsilon }}{2}, \end{array}$$

here

(44) $$\begin{array}{c} \epsilon =\left(b_{42}-\chi_{m}\mu \right)b_{24}-D_{m}\mu \left(b_{22}+D_{c}\mu \right). \end{array}$$

The other two eigenvalues are

(45) $$\begin{array}{c} \sigma_{4}=-b_{33}-\mu D_{f},\\ \sigma_{5}=-b_{55}-\mu D_{e}. \end{array}$$

In the forthcoming remark, explicit representations for all possible *σ*'s are explored for direct comparison between conditions of the stability of the linearized PDE (8) and ODE (15) systems.

**Remark 5**

Similarly to criteria for ODE the stability for PDE, requires that real parts of the all *σ*'s to be negative. Under the natural constraints on the parameters of our original model *b*_11_, *b*_55_, *b*_22_, and *b*_42_ (see Remark 4) we already have *σ*_1_ < 0,*σ*_4_ < 0 and *σ*_5_ < 0. Therefore, our criteria for PDE stability reduce to conditions as follows:

(46) $$\begin{array}{c} b_{22}+D_{c}\mu +D_{m}\mu >0,\;\epsilon <0. \end{array}$$

It is obvious to see that if both inequalities hold, then *σ*_2_  and *σ*_3_ are negative. Since stability of the ODE system forces *b*_24_ > 0 and *b*_42_ < 0, these two inequalities for PDE stability hold for any *χ*_*m*_ > 0, *D*_*m*_ > 0,*D*_*c*_ > 0, *μ* > 0.

From the above arguments it follows that if the ODE system is stable, then $\overset{-}{v}\left(x,t\right)$ are converging to zero as time goes to infinity for any eigenfunction *ϕ*_*n*_. Therefore, since the *ϕ*_*n*_(*x*) is complete in *L*_2_ space, one can conclude that the stability of the linearized PDE system (8) in *L*_2_ space follows from the stability of the ODE system (15).

As expected, the ODE stability and PDE stability are different. Let *D*_*m*_ = *D*_*c*_ *χ*_*m*_ = 0, then the first 5 eigenvalues of the PDE and ODE have the same sign. By definition of our original model *σ*_1_, *σ*_2_, and *σ*_5_ are all negative. Assume *b*_33_ > 0 (in some sense reactive terms has stabilizing effect, with respect to *U*_*e*_), then *σ*_4_ < 0. However, now if one lets *f*_2_ *λ*_3_ > *f*_3_ *λ*_2_ *c*_*e*_, which means that inflammatory macrophages dominate the regulatory macrophages, then *b*_24_ < 0 causing *σ*_3_ > 0, and consequently the ODE system (15) is unstable. For the same set of the coefficients *b*'s and given *μ* > 0, it is not difficult to find sufficient condition on *D*_*m*_, *D*_*c*_, and *χ*_*m*_   such that *σ*_3_ < 0, which guarantee stability of the equilibrium state *U*_*e*_. For example, any set with the same coefficients *b*'s with

(47) $$\begin{array}{c} D_{m}D_{c}>\left(b_{42}-\chi_{\mu }\right)b_{24}/\mu \end{array}$$

will have a real part of the *σ*_3_ < 0 and consequently the solution of the corresponding IBVP with initial function to be *ϕ*_*μ*_(*x*)(*u*_1_,…, *u*_5_) will be vanishing at time infinity. Condition (47) contains the following pattern in the biological interpretation. Assume that inflammatory macrophages dominate the regulatory macrophages and are characterized by the coefficient *b*_24_ = −(*f*_2_ *λ*_3_ − *f*_3_ *λ*_2_ *c*_*e*_) < 0. Then for any given value *b*_24_ if mobility of the macrophages and diffusion of the chemoattractant is high enough in comparison to the coefficient *b*_24_, then *U*_*e*_ is stable for the class of perturbation which corresponds to eigenfunction *ϕ*_*μ*_. In less strict wording, *the system can be cleaned from dead cells by high “mobility/diffusivity” of the macrophages with respect to chemoattractant.* This indicates vital impact of the key parameters *D*_*m*_, *D*_*c*_, and *χ*_*m*_   on “inflammatory” behavior both in space and in time of the system perturbed from equilibrium.

Obtained conclusion depends on *μ* and can be applied only if initial data is proportional to *ϕ*_*μ*_. If in the Fourier extension of the initial data all coefficients are nonzero, then the sufficient condition for stability is the same as for ODE system.

In the next section, we will analyze conditional stability of the IBVP for (8) under assumption that $\overset{-}{v}\left(x,t_{0}\right)\;$ has zero average:$\int_{}^{}\overset{-}{v}\left(x,t_{0}\right)dx=0$. We will derive conditions on the coefficient of the system (8) such that the *L*_2_ norm of the solution is bounded by the *L*_2_ norm of the initial data, or it converges to zero at time infinity depending on the conditions on coefficients. Those conditions will depend only on coefficients of the model and Poincare constant (*C*_*p*_), which in turn depends only on the geometry of the domain. We will also show that there exists a specific initial distribution such that the corresponding IBVP solution is vanishing at time infinity while the corresponding solution of the ODE is unbounded at time infinity.

## 5. Stability of Equilibrium in the Linearized PDE System without ODE Stability

Let us rewrite the linearized system (8) as follows:

(48) ∂d∂t=Dd∇2d−f0λ1med−b14m,

(49) λ1∂c∂t=λ1Dc∇2c−b21λ1d−b22λ1c−b24λ1m,

(50) λ1∂f∂t=Dfλ1∇2f−χfλ1∇2c −b32λ1c−b33λ1f−b34λ1m,

(51) λ1∂m∂t=Dmλ1∇2m−χmλ1∇2c −b42λ1c−b44λ1m,

(52) λ1∂e∂t=Deλ1∇2e−χe1λ1∇2f −χe2λ1∇2c−b53λ1f−b55λ1e.

Next multiplying equations (48) by *d*, (49) by *c*, (50) by *f*, (51) by *m*, and (52) by *e* correspondingly and integrating by parts, one can easily get

(53) 12∂∂t∫d2=−∫Dd(∇d)2−f0λ1med2−b14md,

(54) λ12∂∂t∫c2=−∫λ1Dc(∇c)2−b21λ1dc−b22λ1c2−b24λ1mc,

(55) λ12∂∂t∫f2=−∫Dfλ1(∇f)2+Φ(c,f) −b32λ1cf−b33λ1f2−b34λ1mf,

(56) λ12∂∂t∫m2=−∫Dmλ1(∇m)2+Φ(c,m)  −b42λ1cm−b44λ1m2,

(57) λ12∂∂t∫e2=−∫Deλ1(∇e)2+Φ(f,e) +Φ(c,e)−b53λ1fe−b55λ1e2.

Here, Φ(*f*, *e*) = *χ*_*e*1_ *λ*_1_∇*f*∇*e*, Φ(*c*, *f*) = *χ*_*f*_ *λ*_1_∇*c*∇*f*, Φ(*c*, *m*) = *χ*_*m*_ *λ*_1_∇*c*∇*m*, Φ(*c*, *e*) = *χ*_*e*2_ *λ*_1_∇*c*∇*e*.

Adding LHS and RHS of the equations above: (53)+(54)+(55)+(56)+(57) and applying the Poincare inequality to the terms ∫(∇*u*)^2^ *dx* such that for *C*_*p*_ = *C*_*p*_(*Ω*) > 0,

(58) $$\begin{array}{c} C_{p}\int_{\Omega }^{}u^{2}dx\leq \int_{\Omega }^{}{\left(\nabla u\right)}^{2}dx+{\left(\int_{\Omega }^{}udx\right)}^{2}, \end{array}$$

one can easily get

(59) 12[∫d2+λ1(c2+f2+m2+e2)]t ≤−∫[B(d,m)+B(c,d)+B(c,m)   +B(c,f)+B(f,m)+B(f,e)]  −∫[B(∇c,∇m)+B(∇c,∇f)   +B(∇e,∇c)+B(∇e,∇f)]  +C[(∫d)2+(∫c)2+(∫f)2+(∫m)2+(∫e)2],

where the bilinear forms are

(60) $$\begin{array}{c} B\left(d,m\right)=0, \end{array}$$

(61) $$\begin{array}{c} B\left(c,d\right)=\lambda_{1}\left[\left(\frac{1}{6}D_{c}C_{p}+b_{22}\right)c^{2}+b_{21}dc+\left(f_{0}+D_{d}C_{p}\right)d^{2}\right], \end{array}$$

(62) B(c,m)=λ1[(16DcCp+b22)c2+b2,4cm   +(13DmCp+b44)m2]

(63) $$\begin{array}{c} B\left(f,m\right)=\lambda_{1}\left[\left(\frac{1}{4}D_{f}C_{p}+b_{33}\right)f^{2}+b_{34}cf+\frac{1}{3}D_{m}C_{p}m^{2}\right], \end{array}$$

(64) $$\begin{array}{c} B\left(c,f\right)=\lambda_{1}\left[\frac{1}{6}D_{c}C_{p}c^{2}+b_{32}cf+\frac{1}{3}D_{f}C_{p}f^{2}\right] \end{array}$$

(65) $$\begin{array}{c} B\left(f,e\right)=0, \end{array}$$

(66) $$\begin{array}{c} B\left(\nabla c,\nabla m\right)=\lambda_{1}\left[\frac{1}{6}D_{c}{\left(\nabla c\right)}^{2}-\chi_{m}\nabla c\nabla m+\frac{1}{3}D_{m}{\left(\nabla m\right)}^{2}\right], \end{array}$$

(67) $$\begin{array}{c} B\left(\nabla c,\nabla f\right)=\lambda_{1}\left[\frac{1}{6}D_{c}{\left(\nabla c\right)}^{2}-\chi_{f}\nabla c\nabla f+\frac{1}{4}D_{f}{\left(\nabla f\right)}^{2}\right], \end{array}$$

(68) $$\begin{array}{c} B\left(\nabla f,\nabla e\right)=\lambda_{1}\left[\frac{1}{4}D_{f}{\left(\nabla f\right)}^{2}-\chi_{e2}\nabla f\nabla e+\frac{1}{2}D_{e}{\left(\nabla e\right)}^{2}\right], \end{array}$$

(69) $$\begin{array}{c} B\left(\nabla c,\nabla e\right)=\lambda_{1}\left[\frac{1}{6}D_{c}{\left(\nabla c\right)}^{2}-\chi_{e1}\nabla c\nabla e+\frac{1}{2}D_{e}{\left(\nabla e\right)}^{2}\right]. \end{array}$$

Imposing assumptions that all bilinear forms above are positively defined, one can then conclude that the system is stable. Below, we formulate a sufficient condition for the solution to be stable in *L*_2_ space. The formulation of the assumptions is presented in terms of the parameters of the original system where biological meanings are more evident.

**Condition 1**

If

(70) $$\begin{array}{c} {\left[\left(\frac{1}{6}D_{c}C_{p}+f_{3}\lambda_{2}m_{e}+f_{4}\right)\left(f_{0}+D_{d}C_{p}\right)\right]}^{1/2}\geq \frac{1}{2}f_{1}, \end{array}$$

then *B*(*c*, *d*) ≥ 0.

**Condition 2**

If

(71) [(14DfCp−[a2(1−2feF0)+a12ce−a3])13DmCp]1/2 ≥12a1λ1,

then *B*(*f*, *m*) ≥ 0.

Taking into account actual values for equilibriums *c*_*e*_ and *f*_*e*_ of the inflammatory equilibrium, one can reduce (71) to an inequality, which is easy to interpret.

Namely, assume that

(72) $$\begin{array}{c} {\left[\frac{1}{4}D_{f}D_{m}C_{p}+\sqrt{A}D_{m}\right]}^{1/2}\geq \frac{1}{2}a_{1}\lambda_{1}, \end{array}$$

then *B*(*f*, *m*) ≥ 0. From the previously mentioned,

(73) A=(a2−a3+a12(a0a11))2 +4(a2F0)a1λ1f4f0λ1a0−a11f1f~0λ3f0λ1(f2λ3a11−f3a0λ2).

Due to the assumption (12), parameter *A* is well defined for all values of the coefficients of the original model. Biological meaning of constraint (12) was explained in Remark 3, and it is necessary for the existence of the inflammatory equilibrium. What we want to point out here is that for any set of the parameters there exist large enough *diffusive* constants *D*_*m*_ and *D*_*f*_ that inequality (72) holds, and consequently bilinear form *B*(*f*, *m*) ≥ 0.

**Condition 3**

If

(74) [(16DcCp+(f3λ2me+f4))(13DmCp+(a11me−a0))]2 ≥12[f2λ3−f3λ2ce],

then *B*(*c*, *m*) ≥ 0. For well posedness of the RHS in inequality (74), assume that

(75) (13DmCp+(a11me−a0)) =13DmCp+a11f4f0λ1a0−a11f1f~0λ3f0λ1(f2λ3a11−f3a0λ2)−a0≥0.

We rewrite the above inequality in terms of the parameters of the original model to point out that for any given set of the parameters, there exists big enough coefficient *D*_*m*_, characterizing macrophages mobility, such that inequality (74) holds.

**Condition 4**

If

(76) $$\begin{array}{c} {\left[\frac{1}{6}D_{c}C_{p}\frac{1}{4}D_{f}C_{p}\right]}^{1/2}\geq \frac{1}{2}a_{12}f_{e}, \end{array}$$

then *B*(*c*, *f*) ≥ 0.

**Condition 5**

If

(77) $$\begin{array}{c} {\left[\frac{1}{6}D_{c}\frac{1}{3}D_{m}\right]}^{1/2}\geq \frac{1}{2}\chi_{m}, \end{array}$$

then *B*(∇*c*, ∇*m*) ≥ 0.

**Condition 6**

If

(78) $$\begin{array}{c} {\left[\frac{1}{6}D_{c}\frac{1}{4}D_{f}\right]}^{1/2}\geq \frac{1}{2}\chi_{f}, \end{array}$$

then *B*(∇*c*, ∇*f*) ≥ 0.

**Condition 7**

If

(79) $$\begin{array}{c} {\left[\frac{1}{4}D_{f}\frac{1}{2}D_{e}\right]}^{1/2}\geq \frac{1}{2}\chi_{e2}, \end{array}$$

then *D*(∇*f*, ∇*e*) ≥ 0.

**Condition 8**

If

(80) $$\begin{array}{c} {\left[\frac{1}{6}D_{c}\frac{1}{2}D_{e}\right]}^{1/2}\geq \frac{1}{2}\chi_{e1}, \end{array}$$

then *D*(∇*c*, ∇*e*) ≥ 0.

We now assume that ∫ for all five components *d*(*x*, 0), *c*(*x*, 0), *f*(*x*, 0), *m*(*x*, 0), and *f*(*x*, 0) is equal to 0 (initial data are orthogonal to 1. Then due to no-flux Neumann condition on the boundary for all times,

(81) $$\begin{array}{c} \int_{U}^{}d=\int_{U}^{}c=\int_{U}^{}f=\int_{U}^{}m=\int_{U}^{}e=0. \end{array}$$

Therefore, the above Conditions (1–8) guarantee Lyapunov stability of the linearized system. If further for the same class of initial data we in addition assume strict inequalities in (70)–(76), then system will be asymptotically stable, and *L*_2_ norm of the solution will exponentially converge to zero as time goes to infinity.

Here, we do not assume the ODE stability conditions of the equilibrium in this section. It will be easy to construct a specially inhomogeneous solution of the initial-boundary value problem (IBVP) so that the solution of corresponding ODE for $V=\int_{}^{}\overset{-}{v}\left(x,t\right)dx$ is identically zero, where the PDE solutions can be either stable or unstable by adjusting certain parameters. Indeed, let the domain be a segment [0, *π*] and as in (32), with *ϕ* = cos⁡⁡*x*. Then, in as Section 4, in order for *v*(*x*, *t*) to be a solution of corresponding IBVP it is necessary and sufficient that *σ* to be a root of the characteristic polynomial equation *P*(*σ*) = 0 in (39). To see Conditions (1–8) are essential, we show an example of the system with: (1) Conditions (1–8) are all met, and (2)*P*(*σ*) has a positive root in (39). For selected domain, assume Poincare constant *C*_*p*_ = 1. Assume that all coefficients are such that inequalities in all constraints except inequities in constraints Conditions 3 and 5 are satisfied. Let *b*_22_ ≥ 4/5*D*_*c*_, *a*_11_ *m*_*e*_ ≥ *a*_0_, and 0 > *b*_24_ ≥ −(*D*_*c*_ *D*_*m*_/20)^1/2^. Obviously for these set of the parameter Condition 3 satisfies. Then if $\sqrt{D_{c}D_{m}/60}\;\geq \chi_{m}$ then Condition 5 holds and consequently *v*(*x*, *t*) → 0 as *t* → *∞*. Furthermore, it is not difficult to see that if *b*_22_ = 4/5*D*_*c*_, and *b*_24_ = −(*D*_*c*_ *D*_*m*_/20)^1/2^ then in (42) is positive provided

(82) $$\begin{array}{c} \left(\chi_{m}-b_{42}\right)\;{\left[\frac{D_{c}D_{m}}{20}\right]}^{1/2}\;-\frac{9}{5}D_{m}D_{c}\;>0.\;\; \end{array}$$

Inequality in (82) holds if

(83) $$\begin{array}{c} \chi_{m}>\sqrt{102D_{c}D_{m}} \end{array}$$

Consequently, Condition (83) holds then ||*v*(*x*,*t*)||_*L*_2__ → *∞* as *t* → *∞*. Comparing stability in (77) and instability in (83), the conditions are optimal unto discrepancy in coefficients. In the next remark, we want to highlight the impact of the diffusive parameter and chemotactic coefficients on the stability of the inflammatory equilibrium *U*_*e*_.

**Remark 6**

In all above eight conditions inequalities hold for big enough values of diffusive coefficients *D*'s. This highlights the importance of the spatial distribution of the perturbation for the equilibrium. The major meaning of these condition is that for any set of the parameters if diffusivity coefficients are big enough then *U*_*e*_ is stable. Another key parameter, which characterizes the behavior of the spatial distribution of the system is the chemotactic coefficient. From the example above, one can see that if the chemotactic sensitivity coefficient *χ* is relatively bigger than the diffusivity characteristic of the process, then *U*_*e*_ is unstable. At the same time if it is relatively smaller, as in inequalities (77)–(80), then the inflammatory equilibrium is stable.

## 6. Conclusion and Discussion

To quantitatively study the processes governing inflammatory and fibrotic reactions against foreign bodies, we have built a mathematical model with the capability to predict the trends of macrophage migration, ECM production, and chemoattractant regulation by macrophages in these fibrotic reactions. The initiations of reactions are digestions of debris which are the natural responses of the immune system to damaged cells and tissues due to the implantation process. Our model is built based principally on biochemical mechanisms, and it has served its purpose in providing trends of reactions. The model is expressed by a system of partial differential equations with no flux boundary conditions.

We have considered an equilibrium state of the system and its stability conditions. We have provided a mathematical proof that when this equilibrium is stable in the corresponding ODEs, then it is also stable for the full system in *L* ^2^(*Ω*). However, a system with a parameter set can be conditionally stable in the PDE sense when its ODE system is not necessarily stable. We provided some exclusive conditions for this to happen. These conditions correspond with feasible biological conditions, where the percentage of regulatory macrophages dominates that of the inflammatory macrophages.

We mention here that the system has infinitely many equilibria, all except for one containing at least one free parameter in it. The one under discussion here is called the interior equilibrium as it has 5 nonzero components. This particular equilibrium corresponds to an inflammatory state of the healing process, whose instability is an indicator of three possible dynamics: (1) best case scenario, returning to the healthy state; (2) uncertain development, transition to another “abnormal” equilibrium; (3) acute inflammatory response (worst case scenario), perturbations tend to infinity.

Our main mathematical result indicates that the inflammatory state's stability mainly depends on the reaction dynamics and even that small spatial diffusion and big chemotaxis cannot destabilize the equilibrium which is stable in the reaction-only system. However, if the equilibrium is unstable by its reaction-only system, then spatial diffusion over chemotactic effects can help to stabilize the equilibrium if the initial perturbation is subjected to specific constraints. We did not discuss other equilibrium states due to the length of the paper, but there is no mathematical difficultly in accomplishing these tasks.
